# Organic mulches and irrigation affect *Mesocriconema xenoplax* and *Pratylenchus penetrans* under cherry

**DOI:** 10.2478/jofnem-2025-0058

**Published:** 2025-12-14

**Authors:** Thomas Forge, Kirsten Hannam, Shawn Kuchta, Paige Munro, Mehdi Sharifi, Tristan Watson

**Affiliations:** Agriculture and Agri-Food Canada, Summerland Research and Development Centre, Summerland, British Columbia V0H 1Z2, Canada

**Keywords:** compost, irrigation, *Mesocriconema xenoplax*, nematode ecology, nematode management, orchard nematodes, organic mulch, *Pratylenchus penetrans*

## Abstract

*Mesocriconema xenoplax* and *Pratylenchus penetrans* are important plant parasitic nematodes of cherry trees, but little is known of how soil and water management practices affect the buildup of either species in cherry orchards. A split-plot field experiment was initiated in 2014 to compare five soil treatments (untreated control, preplant fumigated, compost, bark chip mulch, compost+bark chip mulch) under drip and microsprinkler irrigation. Plant-parasitic nematode populations were monitored through 2023. The population of *M. xenoplax* was initially detected in only 3% of the 60 plots whereas *P. penetrans* was initially present in all plots. By 2023, *M. xenoplax* were detected in 70% of plots with maximum population density among plots of 834 *M. xenoplax* 100 cm^−1^ soil. *Mesocriconema xenoplax* became more abundant in compost plots and fumigated plots than in untreated plots, and more abundant under drip than microsprinkler irrigation. In contrast, *P. penetrans* were least abundant in compost plots and less abundant under drip than microsprinkler irrigation. The opposing responses of these two nematode species illustrate tradeoffs in pest pressures that can occur with changes in orchard soil and water management practices, obscuring effects of either species on tree growth.

Sweet cherry (*Prunus avium* L) is an important orchard crop in the Okanagan Valley of British Columbia (BC), Canada and adjacent Pacific Northwest (PNW) states of the United States. Two nematode pests of cherry in the region are migratory endoparasitic root-lesion nematodes, primarily *Pratylenchus penetrans*, and the ectoparasitic ring nematode, *Mesocriconema xenoplax* (Forge et al., 2021). A 2012 survey indicated that root-lesion and ring nematodes were present in 98% and 79% of Okanagan Valley cherry orchards, respectively (Forge et al., 2021). The pathogenicity of *P. penetrans* to cherry is well documented (Edgerton and Parker, 1958; Mai et al., 1994; Melakeberhan et al., 2000). *M. xenoplax* is known to be pathogenic to various *Prunus* tree species, including peach (*P. persica*) and plum (*P. domestica*) (Ferris et al., 2004; McKenry, 2022), but its effects on sweet cherry, specifically, have not been documented experimentally.

As interest in regenerative agricultural practices grows, producers of temperate perennial fruit crops are increasingly exploring the use of organic waste amendments such as composts to increase soil organic matter and improve various aspects of soil health, including suppression of soil-borne pests and pathogens (Thoden et al., 2011; Neher et al., 2022).

Previous field-based research has demonstrated that pre-plant amendment of soil with compost can reduce infection of roots by *P. penetrans* for raspberry (Forge et al., 2016) and sweet cherry (Watson et al., 2017, 2018) replanted into *P. penetrans* infested soil. Fertilizer nitrogen inputs and irrigation practices were shown to affect population densities of *M. xenoplax* parasitizing grapevines in BC (Forge et al., 2019), but no previous studies have examined how *M. xenoplax* responds to soil organic matter management.

In 2014, a field experiment was initiated to assess the effects of combinations of a compost amendment (incorporated into soil before replanting), bark chip mulch (applied to soil surface after planting), and alternative irrigation practices (drip vs microspinkler) on early growth of cherry trees replanted into an old orchard site that was uniformly infested with *P. penetrans*. Data on population dynamics of the *P. penetrans* population from 2014 through 2017 indicated that compost amendment reduced infection of roots by *P. penetrans* relative to other soil treatments (ST) (Watson et al., 2017), and drip irrigation reduced *P. penetrans* population densities relative to microsprinkler irrigation (Watson et al., 2018). During the latter part of the 2014–2017 period, small numbers of *M. xenoplax* were found in samples from a small proportion of the plots, but the *M. xenoplax* population data were not analyzed further or reported. The *M. xenoplax* population at the site began to increase in 2017 and through the subsequent years. Objectives of the research described here are to: (i) document the increase in frequency of occurrence and population densities of *M. xenoplax* at the experimental site through 2023; and (ii) assess effects of the ongoing organic mulch and irrigation treatments on population densities of both *M. xenoplax* and *P. penetrans* over the final 5 yr of the study.

## Materials and Methods

### Experimental site and design

The experimental orchard block was located at the Summerland Research and Development Centre in Summerland, British Columbia (49° 34′N, 119° 39′W). The soil is a Skaha loamy sand (Brown Chernozemic soil) with a relatively low water-holding capacity. The block consisted of 12 adjacent 25-m long rows with a 3-m distance between rows that was planted with apple (“Braeburn” variety on M.26 rootstock) for 13 years prior to fall of 2013, when the apple trees were removed and the site was prepared for replanting to sweet cherry (“Skeena” variety on Gisela 6 rootstock) in spring of 2014.

**Figure 1:**
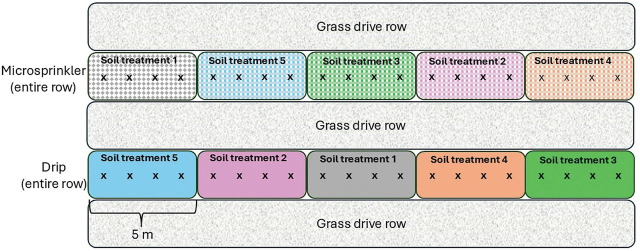
Diagram of one block of the experiment, showing the split-plot experimental design, with entire tree rows allocated either microsprinkler or drip irrigation, and five ST randomly allocated to 5-m long subplots within each row. The four trees per subplot are denoted by “x.” The entire experiment consisted of 6 blocks for a total of 12 rows. ST, soil treatments.

The experiment was set up in a randomized complete block split-plot experimental design. The 12 original rows were grouped into 6 blocks of 2 adjacent rows each, to which the 2 irrigation treatments (drip emitters vs microsprinkler emitters) were randomly allocated (Figure 1). Each row was further divided into five 5-m long subplots to which five different ST were randomly allocated. With a 3-m spacing between rows, each 5-m long plot covered an area of 15 m^2^.

#### Soil Treatments (ST):

The five ST were: (i) Non-treated control; (ii) fumigated; (iii) compost; (iv) bark chips (BC); and (v) compost + BC. The fumigated sub-plots were treated with 33 g · m^−2^ of Basamid granular soil fumigant (Engage Agro Corp. Guelph, ON, Canada): The material was distributed on the soil surface and immediately rototilled into the soil to a depth of 30 cm in October 2013. Non-treated control plots were rototilled at the same time as the fumigated plots.

The compost was a commercially available OMRI-certified material produced from beef-cattle feedlot waste (mixture of bedding and manure), wood sawmill waste, straw, and grape pomace (Bighorn Natural Compost, Big Horn Contracting Ltd., Oliver, BC, Canada) (Table S1 in Supplementary Materials). The compost was initially applied to designated compost and compost + BC plots in spring of 2014, immediately prior to planting. It was applied at a rate of 0.225 m^3^ · plot^−1^ (equivalent to 150 m^3^ compost · ha^−1^) but concentrated on a 1.5 m wide strip centered on the tree row and rototilled into the soil to a depth of 30 cm. In 2016, 2019, and 2021, compost was reapplied to the respective plots at the same rate, but to the soil surface of the 1.5 m wide tree row. In 2016, a municipal yard waste compost produced by the city of Kelowna (GlenGrow compost, Kelowna, BC, Canada) was substituted for the Bighorn compost (Table S1 in Supplementary Materials). Both composts had dry bulk densities of approximately 330 kg · m^−3^ and applications of 150 m^3^ · compost^−1^ · ha^−1^ orchard area were estimated to be 50 Mg dry compost^−1^ · ha^−1^ orchard area. Because compost was applied to the soil surface in all years except 2014, it is hereafter referred to as a mulch.

The BC mulch was applied immediately after planting in 2014. The material was a mixture of shredded and chipped bark and small wood fragments that is a waste product of local sawmills (primarily from lodgepole pine, *Pinus contorta* var *latifolia*). The BC were applied to the soil surface of each plot as a 1.5 m wide strip centered on the tree row to a depth of 5 cm (equivalent to 0.375 m^3^ BC plot^−1^, 250 m^3^ BC ha^−1^). The BC mulch was reapplied to the respective plots in 2016, 2019, and 2021, at rates of 0.375 m^3^ · plot^−1^, 0.2 m^3^ · plot^−1^, and 0.3 m^3^ · plot^−1^, respectively. For plots receiving both compost and BC, the compost was applied first and BC were applied over the compost layer. Nutrient contents of the two composts and BC, and estimated total inputs on a per hectare orchard basis over the course of the study are presented in Table S1 in the Supplementary Materials.

#### Irrigation treatments:

The six drip irrigated rows were each irrigated with two drip lines positioned approximately 15 cm out from the tree row on both sides of the row. The drip lines had 2 L · h^−1^ emitters at 30 cm spacings. The other six rows were each irrigated with 20 L · h^−1^ microsprinklers located between each tree. The microsprinklers delivered water over an approximately 1.5 m band corresponding to the tree row. For both types of irrigation, water was applied daily to supply 100% of the estimated water lost to evapotranspiration the previous day. Evapotranspiration was measured using an electronic atmometer (ETGage Co, Loveland, CO) linked to irrigation valves via a CR10X datalogger (Campbell Scientific, Logan, UT), and a seasonal crop coefficient curve for cherry was used to convert atmometer readings to an estimate of orchard ET as described in Neilsen et al. (2016). Soil moisture contents were monitored using 30-cm Campbell Scientific time domain reflectometry probes (Campbell Scientific, Inc., Logan, UT) positioned approximately 30 cm out from the center of the tree rows. Probes were positioned in three replicate plots of each combination of irrigation and ST, and data were logged hourly over each growing season using a Campbell Scientific datalogger (Campbell Scientific, Inc., Logan, UT). Soil moisture content data were averaged over the months of June, July, and August for presentation (Table S2 in the Supplementary Materials).

The sweet cherry trees (“Skeena” variety grafted onto Gi.6 rootstock) were planted in April, 2014, immediately after incorporation of the compost treatments. Each of the 5-m long sub-plots was planted with four trees with a 1.25 m between-tree spacing. Weed and pest control measures were implemented according to standard production practices (Integrated Fruit Production Guide; https://www.bctfpg.ca/). Herbicides were used to maintain a weed-free strip under the trees that was approximately 1.5 m wide and corresponded with the width of the organic mulch applications and wetting zone of the microspinkler irrigation, and is hereafter referred to as the “root zone.” From 2014 through 2017, soluble N and P were applied to all plots through the irrigation system (fertigated) over 8 weeks each spring to supply 30 g N and 15 g P · tree^−1^. At a planting density of 2,667 trees · ha^−1^, these applications equate to orchard area applications of 80 kg N · ha^−1^ and 40 kg P · ha^−1^. From 2018 through 2023 the entire block was fertilized at similar rates but applied as broadcast granular fertilizer. An exception was 2021 when the compost plots were fertilized at a half rate (15 g N · tree^−1^), because the compost reapplied that year was estimated to provide approximately 15 g N plant available N per tree. Trunk diameters were measured on each of the two middle trees in each plot in 2023 and data were converted to trunk cross-sectional area (TCA) (cm^2^).

### Nematode sampling and extraction

Soil cores were taken for nematode analyses each May and September from 2014 through 2017 as described previously (Watson et al., 2017, 2018). From 2018 through 2023, due to limited resources for nematological analyses, samples were taken each September only. At each sample date, a composite soil sample was taken from each of the 60 plots. Each composite sample comprised six 2-cm diameter × 30-cm deep cores taken from around the two measurement trees in each plot (3 cores · tree^−1^) and combined to form a composite sample representing the plot. To represent the root zone, the three cores from each tree were taken approximately 30 cm out from the trunk as follows: one from along the tree-row axis, one from approximately 45° off the row axis, and one from approximately 90° off the row axis.

Soil samples were passed through an 8-mm opening sieve to remove stones, and root fragments were retained for subsequent extraction of *P. penetrans*. The root fragments were washed over a 1-mm sieve and then approximately 2.0 g samples were cut into approximately 2.0 cm long pieces and subjected to nematode extraction using the shaker agitation technique (Shurtleff and Averre, 2000). After extraction the root fragments were dried and weighed. Nematodes were extracted from 100 cm^−3^ subsamples of the sieved soil using a wet-sieving sucrose centrifugation procedure (Jenkins, 1964). The *P. penetrans* and *M. xenoplax* in each root and soil sample extract were counted using a gridded counting dish on a Meiji Techno TC5000 inverted microscope (Meiji America, Santa Clara, CA). Count data from root and soil extractions were expressed as *P. penetrans* g^−1^ dry root and *M. xenoplax* and *P. penetrans* 100 cm^−3^ soil.

### Data analysis

Chi-square tests were used to determine if the increase in proportion of plots with *M. xenoplax* differed among the 10 treatment combinations, among the five ST (pooled over irrigation treatments), or among the two irrigation treatments (pooled over ST). Because the plots were uniformly infested with *P. penetrans* at the beginning of the experiment (Watson et al., 2017, 2018), the *P. penetrans* data were not subjected to similar Chi-square analyses. A blocked split-plot repeated measures mixed model analysis of variance model (Proc Mixed in SAS, Statistical Analysis Systems, Cary, NC) was used to analyze the effects of irrigation, ST, year, and all interactions on each of the three nematode population density parameters: *M. xenoplax* 100 cm^−3^ soil, *P. penetrans* 100 cm^−3^ soil, and *P. penetrans* g^−1^ root. Irrigation treatments, which were applied to entire rows, were designated as whole-plot treatments, and the ST, which were applied to the five plots within each row, were designated as sub-plot treatments in the model. Block was treated as a random factor and repeated measures were modeled using the autoregressive covariance structure. These analyses were limited to the 5 yr of 2019 through 2023 in which *M. xenoplax* were present in 50% or more of the plots. Analyses of residual plots indicated that all three nematode parameters displayed heteroscedasticity and final analyses were conducted on log(x + 1) transformed data.

To test for influences of the nematode populations on tree growth in the context of the soil and irrigation treatments, TCA data for 2023 were analyzed using a blocked mixed model with fixed effects of irrigation, ST and the irrigation × ST interaction, and with nematode population parameters included as covariates, individually and in combinations (Proc Mixed in SAS, Statistical Analysis Systems). Nematode population parameters used in these analyses were population densities averaged over the 10 yr for each plot. These 10-yr averages approximated the area under the population curve and served as relative indicators of cumulative impacts of the populations culminating in 2023 trunk diameters.

## Results

**Figure 2:**
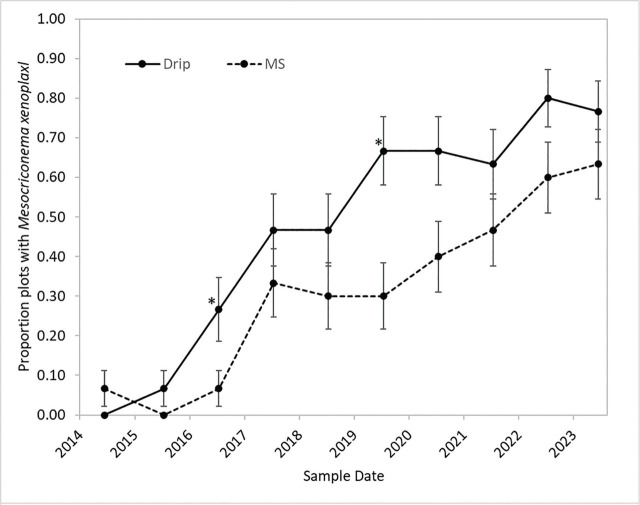
Change through time in the percentage of plots with *M. xenoplax* for drip irrigation (Drip) (30 plots) and MS irrigation (30 plots). Error bars are the standard errors of the proportions (*n* = 30). Asterisks (*) denote individual sample dates at which differences were significant (Chi-square, *P* ≤ 0.05). MS, microsprinkler.

*Mesocriconema xenoplax* were detected at low numbers in only 3% of plots at the start of the study in 2014, but by 2019 and 2023 they were detected in 48% and 70% of plots, respectively (Figure 2). The increase in percentage of plots with *M. xenoplax* did not differ among the 10 ST × irrigation combinations or the five ST (pooled over irrigation treatments) (Chi-square *P* = 0.99 and 0.99, respectively). The increase in percentage infested plots did differ by irrigation treatment (Chi-square *P* < 0.001), however, with a greater percentage of drip irrigated plots infested than microsprinkler irrigated plots across most dates (Figure 2).

*Mesocriconema xenoplax* population densities (nematodes 100 cm^−3^ soil) varied over the last 5 yr of the study (main-factor effect of year *P* = 0.005), with overall means of 42, 47, 52, 83, and 58 *M. xenoplax* 100 cm^−3^ soil for 2019, 2020, 2021, 2022, and 2023, respectively. There were no interactions between year and either irrigation or ST factors (Table 1). Maximum population densities among individual plots were 810, 834, 436, 752, and 648 *M. xenoplax* 100 cm^−1^ soil in 2019, 2020, 2021, 2022, and 2023, respectively. ST affected *M. xenoplax* population densities (main-factor effect *P* = 0.048), with densities in the fumigated and compost plots four to five times greater than in the untreated, bark chip, and compost + bark chip plots (mean comparisons *P* ≤ 0.05; Table 1). Irrigation also affected *M. xenoplax* population densities (*P* = 0.004), with main factor mean population densities under drip irrigation more than two times greater than under microspinkler irrigation (Table 1;Figure 2).

**Table 1:** Main-factor means and mixed model repeated measurement ANOVA summaries for the three nematode population parameters from 2019 through 2023, and cherry tree TCA in 2023.

	***M. xenoplax* 100 cm^−1^ soil**	***P. penetrans* 100 cm^−1^ soil**	***P. penetrans* g^−1^ root**	**2023 TCA cm^2^**
Irrigation (whole-plot) means				
Drip	78*	17*	136*	80
MS	34	24	268	72
ST (subplot) means				
Compost (C)	115a	15b	108	78
BC	20b	25a	181	79
C + BC	26b	25ab	253	78
Fumigated	103a	20ab	293	76
Untreated	19b	19b	176	69
ANOVA summary (*P*-values)				
Irrigation (I)	0.004	0.004	0.039	0.071
ST	0.048	0.017	0.079	0.599
I × ST	0.482	0.267	0.245	0.515
Year (Y)	0.005	<0.001	<0.001	NA
Y × I	0.408	0.036	0.961	NA
Y × ST	0.068	0.178	0.024	NA
Y × I × ST	0.964	0.253	0.369	NA

Main-factor means are for the two irrigation treatments and the five ST. Values within each column of ST means labeled with different letters are significantly different (*P* ≤ 0.05). ANOVA summary *P*-values are for main-factor effects of Irrigation (I), ST and Year (Y), and all interactions.

ANOVA, analysis of variance; BC, bark chips; MS, microsprinkler; ST, soil treatment; TCA, trunk cross-sectional areas.

**Figure 3:**
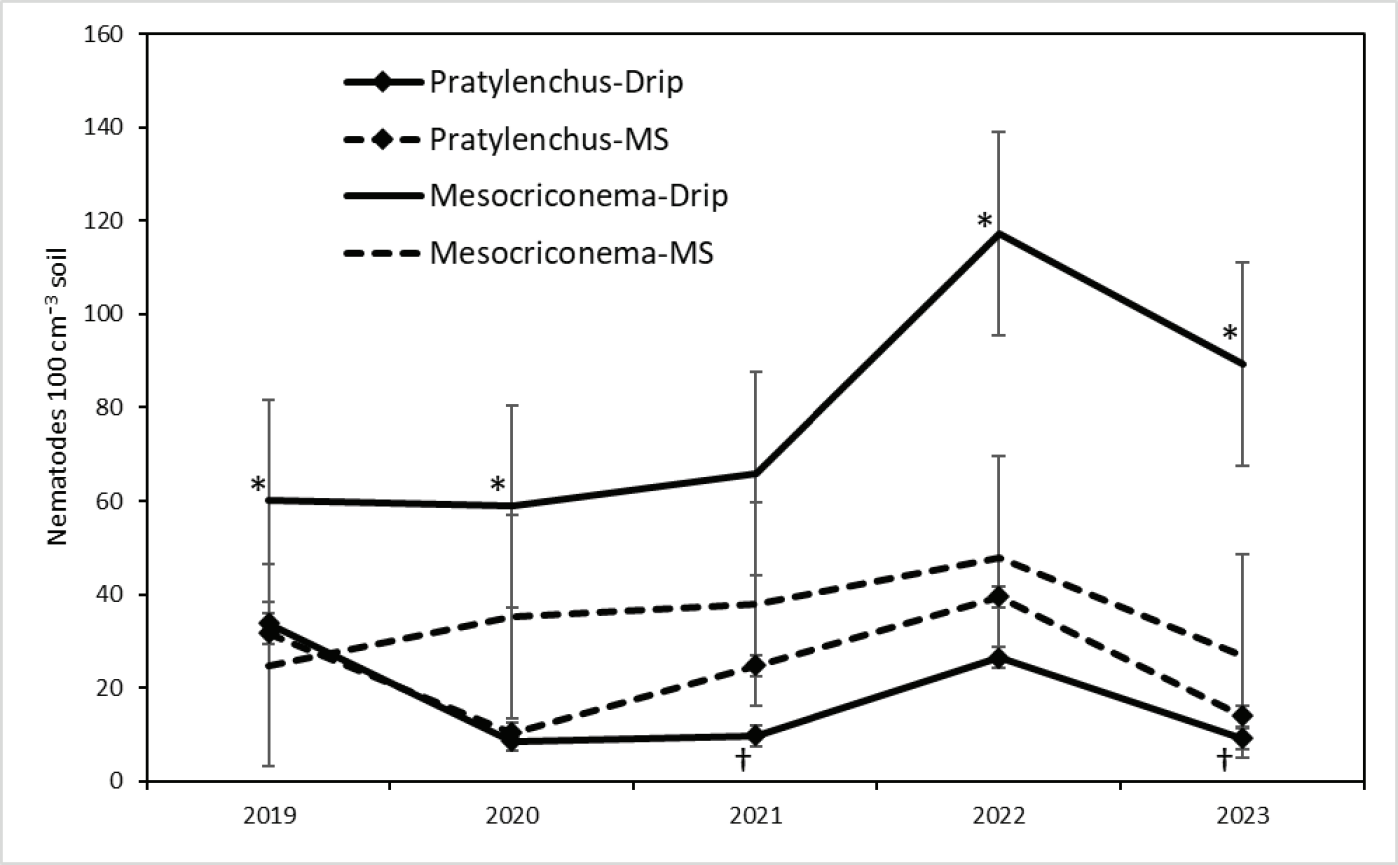
Changes over five annual sample dates in population densities of *M. xenoplax* (100 cm^−1^ soil) and *P. penetrans* (100 cm^−1^ soil) under drip irrigation and MS irrigation in a sweet cherry orchard. Each data point is a main-factor mean of five ST and six blocks (*n* = 30). Error bars are (±) pooled standard errors from mixed model ANOVA of raw data. ANOVA *P*-values (log-transformed data) for irrigation × sample date interaction and irrigation main-factor effects were 0.408 and 0.004 for *M. xenoplax*, and 0.036 and 0.004 for *P. penetrans*, respectively. Individual sample dates at which drip irrigation differs from microsprinkler (*P* < 0.05) are denoted by “*” and “†” for *M. xenoplax* and *P. penetrans*, respectively. ANOVA, analysis of variance; MS, microsprinkler; ST, soil treatment.

*Pratylenchus penetrans* population densities in soil (nematodes 100 cm^−3^ soil) were affected by ST (main-factor effect *P* = 0.017), with the lowest population densities in the compost treatment and the greatest population densities in the bark chip treatment. Population densities in the compost and untreated control plots were lower than in the bark chip and compost + bark chip plots, while population densities in the fumigated plots were intermediate in value and not different from other treatments. Population densities were also affected by the year × irrigation interaction and the main-factor effect of irrigation (*P* = 0.036 and 0.004, respectively); population densities under microsprinkler irrigation tended to be greater than under drip irrigation in four of the 5 yr (Figure 3), with main factor means (averaged over ST and dates) of 24 and 17 *P. penetrans* 100 cm^−3^ soil for microsprinkler and drip irrigation, respectively.

**Figure 4:**
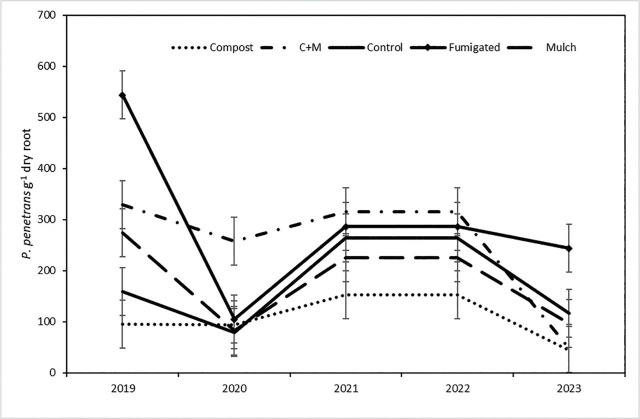
Changes over five annual sample dates in number of *P. penetrans* g^−1^ dry fine root tissue in five ST in a sweet cherry orchard. Each data point is a mean of two irrigation treatments and six blocks (*n* = 12). Error bars are (±) pooled standard errors from mixed model ANOVA of raw data. ANOVA *P*-value (log-transformed data) for the ST × sample date interaction was 0.024. ANOVA, analysis of variance; ST, soil treatment.

Population densities of *P. penetrans* in roots (nematodes g^−1^ dry root) were approximately two times greater under microsprinkler irrigation than drip irrigation (Table 1). The year × ST interaction (*P* = 0.024) reflected considerable fluctuations between years for most of the treatments (Figure 4). The notable exception was the compost only treatment, under which population densities in roots were the most stable and lowest of all treatments at most sample dates. In contrast, population densities in the compost + bark chip treatment were maintained at about 300 *P. penetrans* g^−1^ root from 2019 through 2022 and then dropped in 2023 to a similar value as the compost treatment. The fumigated treatment appeared to have the most variable populations, dropping from about 550 *P. penetrans* g^−1^ root in 2019 to about 100 *P. penetrans* g^−1^ root in 2020 and then stabilizing at about 280 g^−1^ root in the three subsequent years.

### Relationships between nematode population parameters and tree growth:

Analysis of variance of 2023 tree TCA, without nematode parameters included as covariates, indicated a weak effect of irrigation (*P* = 0.071) but not ST or irrigation × ST interaction (Table 1). None of the nematode parameters had significant effects when included as covariates in the ANCOVA of TCA, nor did they substantially alter effects of the fixed factors on TCA (data not shown).

## Discussion

*Mesocriconema xenoplax* became more abundant under the compost and fumigation treatments than under the other three ST, and under drip irrigation than under microsprinkler irrigation. As the proportion of plots with *M. xenoplax* increased through time, we considered that the observed differences in population densities could have been an artifact of treatment-biased colonization of the plots by *M. xenoplax*. Our chi-square analyses indicated, however, that the increase in proportion of plots with *M. xenoplax* did not differ among the ST or combinations of soil and irrigation treatments, indicating that the differential *M. xenoplax* population growth was not an artifact of treatment-biased colonization. We propose that at the time of planting the *M. xenoplax* population was already distributed patchily across the site, but at sub-detectable population densities in many plots. After planting a favorable host (cherry), this initially small population began to increase more rapidly in the compost and initially fumigated plots than in other ST plots, and under drip irrigation than under microspinkler irrigation, resulting in the appearance of a greater proportion of plots with the nematode. The site was an apple orchard for 13 yr prior to being planted with cherry in 2014. A recent survey study found *M. xenoplax* in approximately 50% of Okanagan Valley apple orchards, but at considerably lower population densities on average (9 *M. xenoplax* 100 cm^−3^) than in cherry orchards (81 *M. xenoplax* 100 cm^−3^) and vineyards (103 *M. xenoplax* 100 cm^−3^) (Forge et al., 2021). This pattern of occurrence suggests that apple is not a favorable host for the nematode, but that populations can persist in apple orchards at low densities. It seems likely that *M. xenoplax* populations are often overlooked when apple orchards are converted to cherry orchards or vineyards in the Okanagan Valley.

The increase in *M. xenoplax* population densities in compost plots contrasts with *P. penetrans*, which had the smallest population densities in the compost plots, although they were not significantly less than in untreated plots through years of the study presented here. Suppression of *P. penetrans* in compost-amended soil has been observed in previous shorter-term field studies (Forge et al., 2016; Watson et al., 2017, 2018), and greenhouse experiments indicated that compost-driven suppression of *P. penetrans* can vary among composts and soils (Watson et al., 2019). A review of a wide range of previous studies of effects of composts on plant-parasitic nematodes reported that compost amendments are nearly as likely to increase plant-parasitic nematode populations as to suppress them (Thoden et al., 2011). Our study underscores that review by demonstrating differential responses of two different nematode species to the same compost treatments in the same long-term field experiment.

Compost-induced suppression of *P. penetrans* and other nematodes in other production systems has been ascribed to enhanced activity of antagonists such as predacious nematodes and nematode-trapping fungi (Timper, 2014; Watson et al., 2017, 2018; Neher et al., 2022), although increases in biological suppressiveness have not been clearly demonstrated (Timper, 2014). Reasons for the increase in *M. xenoplax* in compost-amended soil are unclear. One possible explanation is that nutrient enhancement of the compost amended soil and cherry tree roots could have allowed for greater *M. xenoplax* fecundity despite any enhancement of biological control processes. Another possibility is that key predators, parasites, or pathogens of *M. xenoplax*, specifically, are themselves suppressed by a compost-associated factor such as greater nitrogen availability. Elucidating the mechanisms of the *M. xenoplax* increase is worthy of further investigation, but beyond the scope of data collected in this experiment.

Application of the bark chip mulch represented considerable total organic matter inputs to the soil ecosystem. However, it did not result in similar effects as compost for either nematode species, and for combined applications (compost + bark chip treatment) it seemed to negate the compost effects. Given the very high C:N ratio of BC and the tendency to drive N immobilization, it would be expected to have very different effects on the overall soil ecology than compost. Unlike BC, composts are also precolonized by an abundance of microbes, including populations known to have plant health-promoting and biocontrol effects (e.g. Neher et al., 2022).

The increase in *M. xenoplax* populations in fumigated soil relative to untreated soil a few years after fumigation is consistent with observations on the *P. penetrans* population in the first 3 yr of this experiment (Watson et al., 2018) and for other nematode species in other production systems. One working hypothesis is that fumigation also eliminates natural enemies of the plant-parasitic nematodes, allowing for more rapid return population growth of the plant-parasitic nematodes. Alternatively, improved root growth in fumigated plots could also have facilitated more rapid buildup of the *M. xenoplax* population in fumigated plots relative to other ST. Earlier publications reported greater fine root abundance in fumigated soil than in other ST (Watson et al., 2017), and in drip irrigated plots than in microsprinkler irrigated plots (Watson et al., 2018), in the initial years of the study.

The *M. xenoplax* and *P. penetrans* populations also responded in opposite trajectories to the irrigation treatments, with *M. xenoplax* increasing under drip relative to microsprinkler irrigation, whereas *P. penetrans* decreased under drip relative to microsprinkler irrigation. Although similar amounts of water were applied through the drip and microsprinkler irrigation treatments in this experiment, drip irrigation resulted in wetter root zone soil on average (Table S2 in the Supplementary Materials; Watson et al., 2018). While microsprinkler irrigation may provide more uniform lateral distribution of water, it is more prone to evaporative loss of water from soil or mulch surfaces before it infiltrates into the soil, resulting in lower average soil moisture contents. We speculate that the differential responses of *P. penetrans* and *M. xenoplax* to these two different irrigation practices relate to their modes of parasitism. As migratory endoparasites, *P. penetrans* in roots may be less affected by soil moisture *per se* than the ectoparasitic *M. xenoplax*, provided the soil does not become sufficiently dry to cause desiccation of the fine roots. Conversely, *M. xenoplax* appears to benefit from consistently higher soil moisture contents. Previous research examining effects of different vineyard irrigation frequencies documented increased growth of *M. xenoplax* in the more frequent irrigation treatment, which also maintained greater soil moisture contents (Forge et al., 2019).

Because *M. xenoplax* population densities varied substantially across the plots, we considered that variation in tree growth could be affected by *M. xenoplax*. However, analysis of covariance, with nematode population parameters as covariates, did not provide clear indications of the influences of either nematode species on tree growth. Because the two nematode species responded in opposing directions to both irrigation and ST, the effects of either species on tree growth was likely masked by effects of the other species in these analyses. These results illustrate the difficulty in relating orchard productivity to plant-parasitic nematode populations under field conditions, particularly when multiple species are present.

In summary, after an apple orchard block was replanted with cherry, *M. xenoplax* was found to increase over 10 yr from below detectable levels to being present in 70% of plots, with some plots harboring population densities in excess of 800 *M. xenoplax* 100 cm^−3^ soil, and with a mean population density across plots of 113 *M. xenoplax* 100 cm^−3^ soil. The *M. xenoplax* population grew to densities greater in drip irrigated rows than in microsprinkler irrigated rows, and in compost-amended and fumigated plots than in untreated plots. These responses were completely opposite to trends for *P. penetrans*, which were less abundant in drip irrigated rows than in microsprinkler irrigated rows, and tended to be less abundant in compost plots than in other treatment plots.

Despite the development of *M. xenoplax* and *P. penetrans* population densities expected to have deleterious impacts on tree growth, we were unable to measure the effects of either nematode species on vegetative tree growth via analysis of covariance, likely because the two species had confounding effects across the plots. The differing responses of these two nematode species to irrigation and soil organic matter management practices suggest that recommending changes in soil organic matter or irrigation management practices to manage plant-parasitic nematodes need to consider species-specific responses to the practices.

## Supplementary Materials

**Table S1:** Macronutrient concentrations and estimates of cumulative macro-nutrient inputs for the compost and bark chip treatments.

	**C (%)**	**N (%)**	**P (%)**	**K (%)**	**Mg (%)**	**Ca (%)**
AWC	18.9	1.22	0.30	0.84	0.55	2.40
YWC	22.5	1.25	0.30	0.98	0.65	2.40
BC	47.6	0.34	n.d.	n.d.	n.d.	n.d.

Cumulative additions, 2014–2023 (Mg/ha^−1^ orchard area)						

Compost (C)	39.6	2.455	0.6	1.75	1.15	4.8
BC	59.5	0.421	n.d.	n.d.	n.d.	n.d.
C + BC	99.1	2.876	n.d.	n.d.	n.d.	n.d.

The AWC was applied in 2014, 2019, and 2021, while the municipal YWC was applied in 2016. Each compost application was at 50 Mg · ha^−1^ orchard area but concentrated in a 1.5 m band centered on the tree row. AWC, agricultural waste compost; BC, bark chips; n.d., not determined; YWC, yard waste compost.

**Table S2:** Volumetric water contents (cm^−3^ H_2_O · cm^−3^ soil) in treatment plots under MS and drip irrigation regimes.

	**Year**				

	**2018**	**2020**	**2021**	**2022**	**2023**
	MS				

Compost (C)	0.179	0.241	0.259	0.280	0.116
BC	0.168	0.183	0.165	0.200	0.082
C + BC	0.179	0.224	0.165	0.208	0.139
Fumigated	0.179	0.185	0.174	0.167	0.096
MS average	0.176	0.208	0.191	0.214	0.108

	Drip				

Compost (C)	0.181	0.249	0.190	0.194	0.149
BC	0.209	0.296	0.259	0.317	0.222
C + BC	0.165	0.215	0.195	0.186	0.127
Fumigated	0.172	0.279	0.228	0.257	0.171
Drip average	0.181	0.260	0.218	0.239	0.167

Values are averages of hourly measurements through June, July, and August of each year in three replicate plots of each irrigation × ST combination. BC, bark chips; MS, microsprinkler; ST, soil treatment.
